# Compact differences of weighted composition operators on the weighted Bergman spaces

**DOI:** 10.1186/s13660-016-1277-8

**Published:** 2017-01-03

**Authors:** Maocai Wang, Xingxing Yao, Fen Chen

**Affiliations:** 1 School of Computer, China University of Geosciences, Wuhan, 430074 China; 2 School of Mathematics and Statistics, Wuhan University, Wuhan, 430072 China

**Keywords:** 47B33, 30D55, 46E15, weighted Bergman space, weighted composition operator, compact difference

## Abstract

In this paper, we consider the compact differences of weighted composition operators on the standard weighted Bergman spaces. Some necessary and sufficient conditions for the differences of weighted composition operators to be compact are given, which extends Moorhouse’s results in (J. Funct. Anal. 219:70-92, 2005).

## Introduction

Let **D** be the open unit disk in the complex plane **C** and **T** the boundary of **D**. Denote by $H(\mathbf{D})$ the space of all holomorphic functions on **D** and by $\mathbf{S}(\mathbf{D})$ the set of all holomorphic self-maps of **D**. Then, for $u\in H(\mathbf{D})$ and $\varphi\in\mathbf{S}(\mathbf{D})$, the weighted composition operator $u C_{\varphi}$ induced by *u* and *φ* is given by

$$ uC_{\varphi}(f)=u\cdot f\circ\varphi,\quad f\in H(\mathbf{D}). $$

When $u\equiv1$, $u C_{\varphi}$ is the composition operator $C_{\varphi}$, in other words, $C_{\varphi}(f)=f\circ\varphi$, $f\in H(\mathbf{D})$; when $\varphi(z)=z$, $u C_{\varphi}$ is the multiplication operator $M_{u}$, *i.e.*, $M_{u}(f)=u\cdot f$, $f\in H(\mathbf{D})$. Broadly, one is interested in extracting properties of $uC_{\varphi}$ acting on a given Banach space of holomorphic functions on **D** (boundedness, compactness, spectral properties, *etc.*) from function theoretic properties of *u* and *φ* and vice versa. In the past several decades, weighted composition operators on various spaces of holomorphic functions have been studied extensively, *e.g.*, [2–7].

As is well known, an early result of Shapiro and Taylor [8] in 1973 showed the non-existence of the angular derivative of the inducing map at any point of the boundary of the unit disk is a necessary condition for the compactness of the composition operator on the Hardy space $H^{2}({\mathbf {D}})$. Later, MacCluer and Shapiro [9] proved that this condition is a necessary and sufficient condition for the compactness of composition operators on the weighted Bergman spaces $A^{p}_{\alpha}({\mathbf {D}})$ $(\alpha>-1)$. Using the Nevanlinna counting function, Shapiro [10] completely characterized those *φ* which induce compact composition operators on the Hardy space $H^{2}({\mathbf {D}})$. With the basic questions such as compactness settled, it is natural to look at the topological structure of composition operators in the operator norm topology and this topic is of continuing interests in the theory of composition operators. Berkson [11] focused attention on the topological structure with his isolation result on $H^{p}({\mathbf {D}})$ in 1981, which was refined later by Shapiro and Sundberg [12], and MacCluer [13]. In [12], Shapiro and Sundberg posed a question: Do the composition operators on $H^{2}({\mathbf {D}})$ that differ from $C_{\varphi}$ by a compact operator form the component of $C_{\varphi}$ in the operator norm topology? While the same question was answered positively on the weighted Bergman spaces [13], this turned out to be not true on the Hardy space [14]. Some other results on differences of weighted composition operators on spaces of holomorphic functions can be found, for example, in [15–21]. In relation with the study of the topological structures, the difference or the linear sum of composition operators on various settings has been a very active topic [13, 17, 22–24]. Recently, Moorhouse [1] characterized completely the compactness for the difference of two composition operators on the Bergman space over the unit disk, and Al-Rawashdeh and Narayan [25] gave a sufficient condition for the same problem on the Hardy space. Here we continue this line to study compact differences of weighted composition operators acting on the standard weighted Bergman spaces.

The standard weighted Bergman space $A^{2}_{\alpha}$ ($\alpha>-1$) is defined as follows:

$$A^{2}_{\alpha}:= \biggl\{ f\in H(\mathbf{D}): \|f \|_{A_{\alpha}}^{2}= \int _{\mathbf{D}}\bigl\vert f(z)\bigr\vert ^{2}\, d { \lambda}_{\alpha}(z)< \infty \biggr\} , $$

where $d{\lambda}_{\alpha}(z)=\frac{1}{\pi}(\alpha+1)(1-| z|^{2})^{\alpha}\, d A(z)$ and *dA* is the area measure on **D**. As is well known, the Bergman space $A^{2}_{\alpha}$ is a reproducing kernel Hilbert space, the reproducing kernel at $z\in\mathbf{D}$ is $K_{z}(w)=\frac{1}{(1-\overline{z}w)^{\alpha+2}}$ and $\frac{1}{\|K_{z}\|_{A^{2}_{\alpha}}}K_{z}\rightarrow0 $ weakly as $|z|\rightarrow1$.

In Section 2 we recall some related facts and results which are needed in the sequel, and then we prove our main results in Section 3. Section 4 deals with the compact perturbations of finite summations of a given weight composition operator.

*Constants.* Throughout the paper we use the letters *C* and *c* to denote various positive constants which may change at each occurrence. Variables indicating the dependency of constants *C* and *c* will be often specified in the parentheses. We use the notation $X\lesssim Y$ or $Y\gtrsim X$ for non-negative quantities *X* and *Y* to mean $X\le CY$ for some inessential constant $C>0$. Similarly, we use the notation $X\approx Y$ if both $X\lesssim Y$ and $Y \lesssim X$ hold.

## Preliminaries

For $1< t<\infty$ and $\xi\in\mathbf{T}$, let $\Delta_{t,\xi}$ be a non-tangential approach region at *ξ* defined by

$$ \Delta_{t,\xi}:=\bigl\{ z\in\mathbf{D}:|z-\xi|\leq t\bigl(1-\vert z \vert \bigr)\bigr\} $$

and $\Gamma_{t,\xi}$ the boundary curve of $\Delta_{t,\xi}$. Clearly $\Gamma_{t,\xi}$ has a corner at *ξ* with angle less than *π*. A function *f* is said to have a non-tangential limit at *ξ*, if $\lim_{z\rightarrow\xi}f(z)$ exists in each non-tangential region $\Delta _{t,\xi}$.

Let *φ* be a holomorphic self-map of **D**. We say that *φ* has a finite angular derivative at $\xi\in\mathbf{T}$, if there exists a point $\eta\in\mathbf{T}$, such that the non-tangential limit as $z\rightarrow\xi$ of the difference quotient $\frac{\eta-\varphi(z)}{\xi-z}$ exists as a finite complex value. Write

$$ \varphi{'}(\xi):=\angle\lim_{z\rightarrow\xi} \frac{\eta-\varphi (z)}{\xi-z} . $$

Denote $F(\varphi):=\{ \xi\in\mathbf{T}: |\varphi{'}(\xi)|<\infty\}$. For $\xi\in F(\varphi)$, by the Julia-Carathéodory theorem in [26], we have

2.1 $$ \bigl\vert \varphi{'}(\xi)\bigr\vert =\lim _{\substack{z\rightarrow\xi\\ z\in\Gamma_{t,\xi}}}\frac{1-|\varphi(z)|^{2}}{1-|z|^{2}} $$

for any $t>1$.

For any $z\in\mathbf{D}$, let $\sigma_{z}$ be the involutive automorphism of **D** which exchanges 0 to *z*, namely,

$$\sigma_{z}(w)=\frac{z-w}{1-\overline{z}w},\quad w\in\mathbf{D}. $$

The pseudo-hyperbolic distance on **D** is defined by

$$\rho(z,w)=\bigl\vert \sigma_{z}(w)\bigr\vert =\biggl\vert \frac{z-w}{1-\overline{z}w}\biggr\vert ,\quad z,w\in\mathbf{D}. $$

Then, for any $z,w\in\mathbf{D}$, it is easy to see that

$$1-\biggl\vert \frac{z-w}{1-\overline{z}w}\biggr\vert ^{2}=\frac {(1-|z|^{2})(1-|w|^{2})}{|1-\overline{z}w|^{2}} $$

and

2.2 $$ 1-\rho(z,w)\leq\frac{1-|z|^{2}}{|1-\overline{z}w|}\leq1+\rho(z,w). $$

Moreover, for any $z\in\mathbf{D}$ and $0< r<1$, let

$$ E_{r}(z):=\bigl\{ w\in\mathbf{D}: \rho(z,w)< r\bigr\} $$

be the pseudo-hyperbolic disk with ‘center’ *z* and ‘radius’ *r*. It is well known that, for given $0< r<1$,

2.3 $$ \frac{1-\rho(z,w)}{1+\rho(z,w)}\leq\frac{ 1-|z|^{2}}{1-|w|^{2}}\leq\frac {1+\rho(z,w)}{1-\rho(z,w)}, \quad w\in E_{r}(z), $$

and

2.4 $$ {\lambda}_{\alpha}\bigl[E_{r}(z)\bigr]\approx\bigl( 1-|z|^{2}\bigr)^{2+\alpha},\quad z\in \mathbf{D}, $$

where the constants in the estimate above depend only on *r* and *α*. In the sequel, we set $\rho(z):=\rho(\varphi_{1}(z),\varphi_{2}(z))$ for the pseudo-hyperbolic distance of $\varphi_{1}(z)$ and $\varphi_{2}(z)$.

The following lemma is cited from [1].

### Lemma 2.1

*For* $\alpha>-1$, *let* *φ* *be a holomorphic self*-*map of* **D** *and* *u* *a non*-*negative*, *bounded*, *and measurable function on* **D**. *Define the measure* $u {\lambda}_{\alpha}$ *by* $u{\lambda}_{\alpha}(E)$ $:= \int_{E}u(z)\, d {\lambda}_{\alpha}(z)$ *on all Borel subsets* $E\subseteq\mathbf{D}$. *If*

$$ \lim_{|z|\rightarrow1}u(z)\frac{1-|z|^{2}}{1-|\varphi(z)|^{2}}=0, $$

*then* $u{\lambda}_{\alpha}\circ\varphi^{-1}$ *is a compact* *α*-*Carleson measure and the inclusion map* $I_{\alpha}:A^{2}_{\alpha }\rightarrow L^{2}(u{\lambda}_{\alpha}\circ\varphi^{-1})$ *is compact*.

For more details as regards Carleson measures, see Section 2.2 in [26].

## Compact difference

Let $\varphi\in\mathbf{S}(\mathbf{D})$ and $u\in H(\mathbf{D})$. If the weighted composition operator $uC_{\varphi}$ is bounded on $A^{2}_{\alpha}$ ($\alpha>-1$), then the adjoint $(uC_{\varphi})^{*}$ of $uC_{\varphi}$ satisfies

$$ (uC_{\varphi})^{\ast}K_{z}(w)=\overline{u(z)}K_{\varphi(z)}(w), \quad z,w\in \mathbf{D}. $$

For $\varphi\in\mathbf{S}(\mathbf{D})$, by the Schwarz-Pick theorem in [26],

3.1 $$ \frac{1-|z|}{1-|\varphi(z)|}< \frac{1+|\varphi(0)|}{1-|\varphi (0)|}< \infty $$

for any $z\in\mathbf{D}$.

The following lemma can be obtained by modifying Lemma 5.1 in [27] (*e.g.*, at the third line on p.2929 in [27]). See also Proposition 3.2 in [28] in a different form for the unit ball case. Here, we give a more elementary proof for convenience.

### Lemma 3.1

*Let* $\varphi_{1}$ *and* $\varphi_{2}$ *be holomorphic self*-*maps of* **D**. *Then*, *for any* $\xi\in F(\varphi_{1})$, *the following holds*:

$$ \lim_{t\rightarrow\infty}\lim_{\substack{z\rightarrow\xi\\ z\in\Gamma _{t,\xi}}} \frac{1-|\varphi_{1}(z)|^{2}}{1-\varphi_{1}(z)\overline {\varphi_{2}(z)}} = \left \{ \textstyle\begin{array}{l@{\quad}l} 1, &\textit{if } \varphi_{1}(\xi)=\varphi_{2}(\xi) \textit{ and } \varphi _{1}{'}(\xi)=\varphi_{2}{'}(\xi), \\ 0,& \textit{otherwise}. \end{array}\displaystyle \right . $$

### Proof

First we notice that

$$\begin{aligned} \frac{1-|\varphi_{1}(z)|^{2}}{1-\varphi_{1}(z)\overline{\varphi _{2}(z)}}&= \frac{1-|\varphi_{1}(z)|^{2}}{1-|z|^{2}}\cdot\frac {1-|z|^{2}}{1-\varphi_{1}(z)\overline{\varphi_{2}(z)}} \\ &=\frac{1-|\varphi_{1}(z)|^{2}}{1-|z|^{2}}\cdot\frac{1}{\frac {1-|\varphi_{1}(z)|^{2}}{1-|z|^{2}} +\frac{\varphi_{1}(z)\overline{(\varphi_{1}(z)-\varphi _{2}(z))}}{1-|z|^{2}}} \\ &=\frac{1-|\varphi_{1}(z)|^{2}}{1-|z|^{2}}\cdot\frac{1}{\frac {1-|\varphi_{1}(z)|^{2}}{1-|z|^{2}}+\triangle(z)}, \end{aligned}$$

where $\triangle(z)=\frac{\varphi_{1}(z)\overline{(\varphi _{1}(z)-\varphi_{2}(z))}}{1-|z|^{2}}$, and

3.2 $$ \liminf_{z\rightarrow\xi}\bigl\vert \triangle(z)\bigr\vert \geq\liminf_{z\rightarrow\xi}\frac{1}{2}\frac{1-|\varphi_{2}(z)|}{1-|z|}. $$

If $\varphi_{2}$ has no finite angular derivative at *ξ*, namely,

$$\liminf_{z\rightarrow\xi}\frac{1-|\varphi_{2}(z)|}{1-|z|}=\infty, $$

then, for any $t>1$, by (3.2), we have

$$\lim_{\substack{z\rightarrow\xi\\ z\in\Gamma_{t,\xi}}} \bigl\vert \triangle (z)\bigr\vert =\infty. $$

If $\varphi_{2}$ has finite angular derivative at *ξ* and $\varphi _{1}(\xi)\neq\varphi_{2}(\xi)$, then it follows clearly that

$$\lim_{\substack{z\rightarrow\xi\\ z\in\Gamma_{t,\xi}}} \bigl\vert \triangle (z)\bigr\vert =\infty. $$

If $\varphi_{2}$ has finite angular derivative at *ξ* and $\varphi _{1}(\xi)=\varphi_{2}(\xi)$, then

$$\begin{aligned} \lim_{\substack{z\rightarrow\xi\\ z\in\Gamma_{t,\xi}}} \bigl\vert \triangle (z)\bigr\vert &=\lim _{\substack{z\rightarrow\xi\\ z\in\Gamma_{t,\xi}}} \biggl\vert \frac{\overline{\xi-z}}{1-|z|^{2}}\varphi_{1}(z) \overline{ \biggl(\frac {\varphi_{2}(\xi)-\varphi_{2}(z)}{\xi-z}-\frac{\varphi_{1}(\xi)-\varphi _{1}(z)}{\xi-z} \biggr)}\biggr\vert \\ &=\frac{t}{2}\bigl\vert \varphi'_{2}(\xi)- \varphi'_{1}(\xi)\bigr\vert . \end{aligned}$$

Thus if $\varphi_{1}(\xi)=\varphi_{2}(\xi)$ and $\varphi'_{1}(\xi )=\varphi'_{2}(\xi)$, we have

$$\lim_{t\rightarrow\infty}\lim_{\substack{z\rightarrow\xi\\ z\in\Gamma _{t,\xi}}} \triangle(z)=0. $$

Otherwise if $\varphi_{1}(\xi)=\varphi_{2}(\xi)$ and $\varphi'_{1}(\xi )\neq\varphi'_{2}(\xi)$, then

$$\lim_{t\rightarrow\infty}\lim_{\substack{z\rightarrow\xi\\ z\in\Gamma _{t,\xi}}} \bigl\vert \triangle(z)\bigr\vert =\infty. $$

Consequently, we get the desired result. □

To further study compact differences of weighted composition operators on $A^{2}_{\alpha}$, we define $F_{u}(\varphi)$ as

$$ F_{u}(\varphi):= \biggl\{ \xi\in\mathbf{T}: \limsup _{z\rightarrow\xi }\bigl\vert u(z)\bigr\vert ^{2} \frac{1-|z|^{2}}{1-|\varphi(z)|^{2}}\neq0 \biggr\} . $$

It is easy to check that $F_{u}(\varphi)\subseteq F(\varphi)$ if *u* is bounded. To avoid the trivial case, in the sequel we assume $F_{u_{i}}(\varphi_{i})\neq\emptyset$, $i=1,2$, *i.e.*, neither $u_{1}C_{\varphi_{1}}$ nor $u_{2}C_{\varphi_{2}}$ is compact on $A^{2}_{\alpha}$.

In the following we take the test functions

$$g_{w}(z):=\frac{(1-|w|^{2})^{\frac{1}{2}}}{(1-\overline{w}z)^{\frac {\alpha+3}{2}}}, \quad w,z\in\mathbf{D}. $$

First note that $\{g_{w}\}$ is bounded in $A^{2}_{\alpha}$. Indeed, note that

$$I_{c}(w)= \int_{\mathbf{D}}\frac{(1-|z|^{2})^{\alpha}}{|1-\overline {w}z|^{\alpha+2+c}}\, dA(z)\approx\frac{1}{(1-|w|^{2})^{c}}, \quad |w|\rightarrow1 $$

for $c>0$ by Lemma 4.2.2 in [29], and then

$$ \|g_{w}\|^{2}_{A^{2}_{\alpha}}=\frac{\alpha+1}{\pi} \int_{\mathbf {D}}\frac{(1-|w|^{2})(1-|z|^{2})^{\alpha}}{|1-\overline{w}z|^{\alpha +3}}\, dA(z)< \infty. $$

Again it is well known that $g_{w}(z)\rightarrow0$ uniformly on any compact subset of **D**, and hence that $g_{w}\rightarrow0$ weakly as $|w|\rightarrow1$.

We now give some necessary conditions for the difference of weighted composition operators to be compact.

### Theorem 3.2

*Let* $\varphi_{1}$, $\varphi_{2}$ *be holomorphic self*-*maps of* **D** *and let* $u_{1}$, $u_{2}$ *be bounded holomorphic functions on* **D** *such that neither* $u_{1}C_{\varphi_{1}}$ *nor* $u_{2}C_{\varphi_{2}}$ *is compact on* $A^{2}_{\alpha}$. *If* $u_{1}C_{\varphi_{1}}-u_{2}C_{\varphi_{2}}$ *is compact on* $A^{2}_{\alpha }$, *then the following statements are true*:

1. $F_{u_{1}}(\varphi_{1})=F_{u_{2}}(\varphi_{2})$.
2. $\angle\lim_{z\rightarrow\xi}|u_{1}(z)-u_{2}(z)|=0$ *for any* $\xi\in F_{u_{1}}(\varphi_{1})$.
3. $\lim_{|z|\rightarrow1} \rho(z) (|u_{1}(z)|^{2}\frac {1-|z|^{2}}{1-|\varphi_{1}(z)|^{2}}+| u_{2}(z)|^{2}\frac {1-|z|^{2}}{1-|\varphi_{2}(z)|^{2}} )=0$.

### Proof

Denote $T:=u_{1}C_{\varphi_{1}}-u_{2}C_{\varphi_{2}}$ for short, and assume that *T* is compact on $A^{2}_{\alpha}$. For $\xi\in F_{u_{1}}(\varphi_{1})$, it is easy to see that

3.3 $$ \lim_{\substack{w\rightarrow\xi\\ w\in\Gamma_{t,\xi}}} \Vert Tg_{\varphi _{1}(w)}\Vert ^{2}_{A^{2}_{\alpha}}=0 $$

for any $t>1$. Using the submean value type inequality in [30] and equation (2.4), then, for a given $0< r<1$,

$$ \Vert Tg_{\varphi_{1}(w)}\Vert ^{2}_{A^{2}_{\alpha}}\ge \int _{E_{r}(w)}\bigl\vert Tg_{\varphi_{1}(w)}(z)\bigr\vert ^{2}\, d{\lambda}_{\alpha}(z)\gtrsim\bigl( 1-\vert w\vert ^{2}\bigr)^{\alpha+2}\bigl\vert Tg_{\varphi_{1}(w)}(w)\bigr\vert ^{2}. $$

So by (3.3)

$$\lim_{\substack{w\rightarrow\xi\\ w\in\Gamma_{t,\xi}}} \biggl(\frac{ 1-|w|^{2}}{1-|\varphi_{1}(w)|^{2}} \biggr)^{\alpha+2} \biggl\vert u_{1}(w)-u_{2}(w) \biggl(\frac{1-|\varphi_{1}(w)|^{2}}{1-{\overline {\varphi_{1}(w)}\varphi_{2}(w)}} \biggr)^{\frac{\alpha+3}{2}}\biggr\vert ^{2}=0 $$

for all $t>1$. Since $u_{1}$, $u_{2}$ are bounded holomorphic functions on **D** and $\xi\in F(\varphi_{1})$, then it follows from our assumption $\xi\in F_{u_{1}}(\varphi_{1})$ that

$$\lim_{t\rightarrow\infty}\lim_{\substack{w\rightarrow\xi\\ w\in\Gamma _{t,\xi}}} \frac{1-|\varphi_{1}(w)|^{2}}{1-\overline{\varphi _{2}(w)}\varphi_{1}(w)}=1, $$

and therefore

$$\lim_{t\rightarrow\infty}\lim_{\substack{w\rightarrow\xi\\ w\in\Gamma _{t,\xi}}} \bigl\vert u_{1}(w)-u_{2}(w)\bigr\vert =0. $$

So (2) is obtained, and thus $F_{u_{1}}(\varphi_{1})\subseteq F_{u_{2}}(\varphi_{2})$ by Lemma 3.1. Similarly, we have $F_{u_{2}}(\varphi_{2})\subseteq F_{u_{1}}(\varphi_{1})$. Thus the proof for (1) is complete.

To prove (3), we assume that there exists a sequence $\{z_{n}\} $ with $|z_{n}|\rightarrow1$ such that

$$\lim_{n\rightarrow\infty}\rho(z_{n}) \biggl(\bigl\vert u_{1}(z_{n})\bigr\vert ^{2}\frac{1-|z_{n}|^{2}}{1-| \varphi _{1}(z_{n})|^{2}}+ \bigl\vert u_{2}(z_{n})\bigr\vert ^{2} \frac{1-| z_{n}|^{2}}{1-|\varphi _{2}(z_{n})|^{2}} \biggr)> 0. $$

Without loss of generality, we may further assume that

$$\bigl\vert u_{1}(z_{n})\bigr\vert ^{2} \frac{1-\vert z_{n}\vert ^{2}}{1-\vert \varphi_{1}(z_{n})\vert ^{2}}\geq \bigl\vert u_{2}(z_{n})\bigr\vert ^{2}\frac{1-\vert z_{n}\vert ^{2}}{1-\vert \varphi_{2}(z_{n})\vert ^{2}} $$

for all *n*. Then

3.4 $$ \limsup_{n\rightarrow\infty}\rho(z_{n})\bigl\vert u_{1}(z_{n})\bigr\vert ^{2} \frac {1-|z_{n}|^{2}}{1-| \varphi_{1}(z_{n})|^{2}}>0. $$

Due to (3.1) and the boundedness of $u_{1}$ on **D**, by passing to a subsequence if necessary, we can suppose that

$$\lim_{n\rightarrow\infty}\rho(z_{n})=a_{0},\qquad \lim _{n\rightarrow\infty }\bigl\vert u_{1}(z_{n})\bigr\vert ^{2}=a_{1}\quad \mbox{and}\quad \lim _{n\rightarrow\infty}\frac{1-\vert z_{n}\vert ^{2}}{1-\vert \varphi_{1}(z_{n})\vert ^{2}}=a_{2} $$

for some constants $a_{0}\in(0,1]$, $a_{1}>0$, $a_{2}>0$. Then $\lim_{n\rightarrow\infty}|u_{2}(z_{n})|^{2}=a_{1}$ by the obtained facts (1) and (2). Actually, we may further assume that

$$ \lim_{n\rightarrow\infty}\frac{1-| z_{n}|^{2}}{1-|\varphi_{2}(z_{n})|^{2}}=a_{3} $$

for some $a_{3}>0$. We put $f_{n}:=K_{z_{n}}/\|K_{z_{n}}\| _{A^{2}_{\alpha}}$, where $K_{z_{n}}$ is the reproducing kernel function at $z_{n}\in \mathbf{D}$ in $A^{2}_{\alpha}$ for each $n\geq1$. So $f_{n}\rightarrow0$ weakly as $n\rightarrow\infty$. We will arrive at a contradiction to the compactness of $u_{1}C_{\varphi _{1}}-u_{2}C_{\varphi_{2}}$ by showing $(u_{1}C_{\varphi_{1}}-u_{2}C_{\varphi_{2}})^{\ast }f_{n}\nrightarrow0$ ($n\rightarrow\infty$) in $A^{2}_{\alpha}$. In fact, notice that

$$\begin{aligned}& \bigl\Vert (u_{1}C_{\varphi_{1}}-u_{2}C_{\varphi_{2}})^{\ast}f_{n} \bigr\Vert ^{2}_{A^{2}_{\alpha}} \\& \quad = \frac{1}{\Vert K_{z_{n}}\Vert ^{2}_{A^{2}_{\alpha}}} \int_{\mathbf{D}} \bigl\vert \overline{u_{1}(z_{n})}K_{\varphi_{1}(z_{n})} -\overline{u_{2}(z_{n})}K_{\varphi_{2}(z_{n})}\bigr\vert ^{2}\, d{\lambda }_{\alpha} \\& \quad \gtrsim \bigl\vert u_{1}(z_{n})\bigr\vert ^{2} \biggl(\frac{1-|z_{n}|^{2}}{1-| \varphi _{1}(z_{n})|^{2}} \biggr)^{\alpha+2}+\bigl\vert u_{2}(z_{n})\bigr\vert ^{2} \biggl( \frac{1-| z_{n}|^{2}}{1-|\varphi_{2}(z_{n})|^{2}} \biggr)^{\alpha+2} \\& \qquad {}-2\bigl\vert u_{1}(z_{n})u_{2}(z_{n}) \bigr\vert \bigl(1-\rho^{2}(z_{n})\bigr)^{\frac{\alpha +2}{2}} \frac{(1-|z_{n}|^{2})^{\alpha+2}}{(1-|\varphi _{1}(z_{n})|^{2})^{\frac{\alpha+2}{2}}(1-|\varphi _{2}(z_{n})|^{2})^{\frac{\alpha+2}{2}}} \\& \quad \geq 2\bigl(1-\bigl(1-\rho^{2}(z_{n}) \bigr)^{\frac{\alpha +2}{2}}\bigr)\bigl\vert u_{1}(z_{n})u_{2}(z_{n}) \bigr\vert \frac{(1-| z_{n}|^{2})^{\alpha +2}}{(1-|\varphi_{1}(z_{n})|^{2})^{\frac{\alpha+2}{2}}(1-|\varphi _{2}(z_{n})|^{2})^{\frac{\alpha+2}{2}}} \end{aligned}$$

for all *n*. Then

$$\liminf_{n\rightarrow\infty}\bigl\Vert (u_{1}C_{\varphi_{1}}-u_{2}C_{\varphi _{2}})^{\ast}f_{n} \bigr\Vert ^{2}_{A^{2}_{\alpha}}\geq2\bigl(1-\bigl(1-a^{2}_{0} \bigr)^{\frac {\alpha+2}{2}}\bigr)a_{1}^{2}(a_{2}a_{3})^{\frac{\alpha+2}{2}}>0. $$

The contradiction implies (3), which completes the proof. □

To give a sufficient condition for the compact difference of weighted composition operators, we need the following fact from [28], pp.95-97: for any $\varepsilon>0$ small enough and $f\in A^{2}_{\alpha}$,

3.5 $$ \int_{\{z\in\mathbf{D}: \rho(z)\leq\varepsilon\}}\bigl\vert f(\varphi _{1})-f( \varphi_{2})\bigr\vert ^{2}\, d{\lambda}_{\alpha} \lesssim\varepsilon^{2}\|f\| ^{2}_{A^{2}_{\alpha}}. $$

To simplify our sufficient condition, we use the following simple lemma.

### Lemma 3.3

*Let* $\varphi_{1}$, $\varphi_{2}$ *be holomorphic self*-*maps of* **D** *and let* $u_{1}$, $u_{2}$ *be bounded holomorphic functions on* **D**. *If*

$$\lim_{z\rightarrow\xi}\bigl\vert u_{1}(z)-u_{2}(z) \bigr\vert =0\quad \textit{for any } \xi\in F_{u_{1}}(\varphi_{1}) \cup F_{u_{2}}(\varphi_{2}) $$

*and*

$$\lim_{|z|\rightarrow1} \rho(z) \biggl(\bigl\vert u_{1}(z) \bigr\vert ^{2}\frac {1-|z|^{2}}{1-|\varphi_{1}(z)|^{2}}+\bigl\vert u_{2}(z)\bigr\vert ^{2}\frac {1-|z|^{2}}{1-|\varphi_{2}(z)|^{2}} \biggr)=0, $$

*then* $F_{u_{1}}(\varphi_{1})=F_{u_{2}}(\varphi_{2})$.

### Proof

If $F_{u_{1}}(\varphi_{1})\neq F_{u_{2}}(\varphi_{2})$, we may assume that $\xi\in F_{u_{1}}(\varphi_{1})$ but $\xi\notin F_{u_{2}}(\varphi_{2})$. Then $\xi\in F(\varphi_{1})$, and $\xi\notin F(\varphi_{2})$ by the assumption $\lim_{z\rightarrow\xi}|u_{1}(z)-u_{2}(z)|=0$ and $\xi\in F_{u_{1}}(\varphi_{1})$. Hence by Lemma 3.1,

$$\lim_{t\rightarrow\infty}\lim_{\substack{z\rightarrow\xi\\ z\in\Gamma _{t,\xi}}} \frac{1-|\varphi_{1}(z)|^{2}}{1-\varphi_{1}(z)\overline {\varphi_{2}(z)}}=0. $$

Note that

$$1-\rho^{2}(z)=\frac{(1-|\varphi_{1}(z)|^{2})(1-|\varphi _{2}(z)|^{2})}{|1-\overline{\varphi_{1}(z)}\varphi_{2}(z)|^{2}}, $$

and (2.2) implies

$$\frac{1-|\varphi_{2}(z)|^{2}}{|1-\overline{\varphi_{1}(z)}\varphi _{2}(z)|}\le2. $$

So

$$\lim_{t\rightarrow\infty}\lim_{\substack{z\rightarrow\xi\\ z\in\Gamma _{t,\xi}}} \rho(z)=1, $$

and then

$$\lim_{t\rightarrow\infty}\lim_{\substack{z\rightarrow\xi\\ z\in\Gamma _{t,\xi}}} \rho(z) \biggl(\bigl\vert u_{1}(z)\bigr\vert ^{2}\frac{1-|z|^{2}}{1-|\varphi _{1}(z)|^{2}}+\bigl\vert u_{2}(z)\bigr\vert ^{2}\frac{1-|z|^{2}}{1-|\varphi _{2}(z)|^{2}} \biggr) \neq0. $$

This leads to a contradiction to the assumption. Thus $F_{u_{1}}(\varphi _{1})=F_{u_{2}}(\varphi_{2})$. □

We are now ready to give our sufficiency theorem.

### Theorem 3.4

*Let* $\varphi_{1}$, $\varphi_{2}$ *be holomorphic self*-*maps of* **D** *and let* $u_{1}$, $u_{2}$ *be bounded holomorphic functions on* **D**. *If the following hold*:

1. $\lim_{z\rightarrow\xi}|u_{1}(z)-u_{2}(z)|=0$ *for any* $\xi \in F_{u_{1}}(\varphi_{1})\cup F_{u_{2}}(\varphi_{2})$ *and*
2. $\lim_{|z|\rightarrow1} \rho(z) (|u_{1}(z)|^{2}\frac {1-|z|^{2}}{1-|\varphi_{1}(z)|^{2}}+| u_{2}(z)|^{2}\frac {1-|z|^{2}}{1-|\varphi_{2}(z)|^{2}} )=0$,

*then* $u_{1}C_{\varphi_{1}}-u_{2}C_{\varphi_{2}}$ *is compact on* $A^{2}_{\alpha}$.

### Proof

Assume that $\{f_{n}\}$ is any bounded sequence in $A^{2}_{\alpha}$ such that $f_{n}\rightarrow0$ ($n\rightarrow\infty$) uniformly on each compact subsets of **D**. Given $\varepsilon>0$, we put

$$Q:=\bigl\{ z\in\mathbf{D}: \rho(z)\leq\varepsilon\bigr\} ,\qquad Q{'}:=\mathbf {D}\backslash Q. $$

Now we can write

3.6 $$ \bigl\Vert (u_{1}C_{\varphi_{1}}-u_{2}C_{\varphi_{2}})f_{n} \bigr\Vert ^{2}_{A^{2}_{\alpha }}= \int_{\mathbf{D}}\vert u_{1}C_{\varphi_{1}}f_{n}-u_{2}C_{\varphi _{2}}f_{n} \vert ^{2}\, d{\lambda}_{\alpha}= \int_{Q}+ \int_{Q{'}} $$

for each *n*.

Let $\chi_{Q{'}}$ be the characteristic function of $Q{'}$, then by the assumption (2),

$$\lim_{|z|\rightarrow1}\chi_{Q{'}} \biggl(\bigl\vert u_{1}(z)\bigr\vert ^{2}\frac {1-|z|^{2}}{1-|\varphi_{1}(z)|^{2}}+\bigl\vert u_{2}(z)\bigr\vert ^{2}\frac {1-|z|^{2}}{1-|\varphi_{2}(z)|^{2}} \biggr)=0. $$

So by Lemma 2.1, for the second term of the right-hand side of (3.6),

$$\begin{aligned}& \int_{Q{'}}\bigl\vert u_{1}f_{n}( \varphi_{1})-u_{2}f_{n}(\varphi _{2}) \bigr\vert ^{2}\,d\lambda_{\alpha} \\& \quad \lesssim \int_{\mathbf{D}}\bigl\vert \chi_{Q{'}}u_{1}C_{\varphi _{1}}(f_{n}) \bigr\vert ^{2}\,d\lambda_{\alpha}+ \int_{\mathbf{D}}\bigl\vert \chi _{Q{'}}u_{2}C_{\varphi_{2}}(f_{n}) \bigr\vert ^{2}\,d\lambda_{\alpha}\rightarrow0 \end{aligned}$$

as $n\rightarrow\infty$. For any $\xi\in F_{u_{1}}(\varphi_{1})$, by the assumption (1), there exists $\delta(\xi)>0$ such that $|u_{1}(z)-u_{2}(z)|<\varepsilon$ whenever $|z-\xi|<\delta(\xi)$. We decompose *Q* into two parts, $Q:=H_{1}+H_{2}$, where $H_{1}:=Q\cap (\bigcup_{\xi\in F_{u_{1}}(\varphi_{1})}\{z\in \mathbf{D}:|z-\xi|<\delta(\xi)\} )$ and $H_{2}:=Q\backslash H_{1}$. Also, for the first term of the right-hand side of (3.6), we have

3.7 $$\begin{aligned}& \int_{Q}\bigl\vert u_{1}f_{n}( \varphi_{1})-u_{2}f_{n}(\varphi_{2}) \bigr\vert ^{2}\,d\lambda_{\alpha} \\& \quad \lesssim \int_{Q}\vert u_{1}\vert ^{2}\bigl\vert f_{n}(\varphi_{1})-f_{n}( \varphi_{2})\bigr\vert ^{2}\,d\lambda_{\alpha}+ \int_{Q}\vert u_{1}-u_{2}\vert ^{2}\bigl\vert f_{n}(\varphi_{2})\bigr\vert ^{2}\,d\lambda_{\alpha} \\& \quad \lesssim \int_{Q}\bigl\vert f_{n}(\varphi_{1})-f_{n}( \varphi_{2})\bigr\vert ^{2}\,d\lambda_{\alpha }+ \int_{Q}\vert u_{1}-u_{2}\vert ^{2}\bigl\vert f_{n}(\varphi_{2})\bigr\vert ^{2}\,d\lambda_{\alpha} \\& \quad \lesssim \int_{Q}\bigl\vert f_{n}(\varphi_{1})-f_{n}( \varphi_{2})\bigr\vert ^{2}\,d\lambda_{\alpha }+\sum _{i=1}^{i=2} \int_{ H_{i}}\vert u_{1}-u_{2}\vert ^{2}\bigl\vert f_{n}(\varphi _{2})\bigr\vert ^{2}\,d\lambda_{\alpha} \\& \quad \lesssim \int_{Q}\bigl\vert f_{n}(\varphi_{1})-f_{n}( \varphi_{2})\bigr\vert ^{2}\,d\lambda_{\alpha }+ \int_{ H_{1}}\vert u_{1}-u_{2}\vert ^{2}\bigl\vert f_{n}(\varphi_{2})\bigr\vert ^{2}\,d\lambda_{\alpha} \\& \qquad {}+ \sum_{i=1}^{i=2} \int_{ H_{2}}\vert u_{i}\vert ^{2}\bigl\vert f_{n}(\varphi _{2})\bigr\vert ^{2}\,d \lambda_{\alpha}. \end{aligned}$$

Note that by (3.5)

$$\int_{Q}\bigl\vert f_{n}(\varphi_{1})-f_{n}( \varphi_{2})\bigr\vert ^{2}\, d\lambda_{\alpha } \lesssim\varepsilon^{2} $$

for all *n*. Also, by the definition of $H_{1}$, we can easily get

$$\int_{ H_{1}}\vert u_{1}-u_{2}\vert ^{2}\bigl\vert f_{n}(\varphi_{2})\bigr\vert ^{2}\, d\lambda_{\alpha }\leq\varepsilon^{2}\Vert C_{\varphi_{2}}f_{n}\Vert ^{2}_{A^{2}_{\alpha }}\lesssim \varepsilon^{2} $$

for all *n* and

3.8 $$ \lim_{|z|\rightarrow1}\chi_{H_{2}}\bigl\vert u_{1}(z)\bigr\vert ^{2}\frac {1-|z|^{2}}{1-|\varphi_{1}(z)|^{2}}=0. $$

We now claim that

3.9 $$ \lim_{|z|\rightarrow1}\chi_{H_{2}}\bigl\vert u_{2}(z)\bigr\vert ^{2}\frac {1-|z|^{2}}{1-|\varphi_{2}(z)|^{2}}=0 $$

and

3.10 $$ \lim_{|z|\rightarrow1}\chi_{H_{2}}\bigl\vert u_{1}(z)\bigr\vert ^{2}\frac {1-|z|^{2}}{1-|\varphi_{2}(z)|^{2}}=0. $$

Indeed, if either (3.9) or (3.10) fails, then we will arrive at a contradiction to (3.8), and thus the desired is obtained. To this end, we assume that there exist some $\eta\in\mathbf{T}$ and a sequence $z_{n} \in H_{2}$ satisfying $z_{n}\rightarrow\eta$ such that

3.11 $$ \lim_{n\rightarrow\infty}\bigl\vert u_{2}(z_{n}) \bigr\vert ^{2}\frac {1-|z_{n}|^{2}}{1-|\varphi_{2}(z_{n})|^{2}}>0, $$

or

3.12 $$ \lim_{n\rightarrow\infty}\bigl\vert u_{1}(z_{n}) \bigr\vert ^{2}\frac {1-|z_{n}|^{2}}{1-|\varphi_{2}(z_{n})|^{2}}>0. $$

If (3.11) holds, then $\eta\in F_{u_{2}}(\varphi_{2})$. Thus $\eta \in F_{u_{1}}(\varphi_{1})$ due to the fact that $F_{u_{2}}(\varphi_{2})=F_{u_{1}}(\varphi_{1})$ by Lemma 3.3. If (3.12) holds, then

$$\lim_{n\rightarrow\infty}\bigl\vert u_{1}(z_{n})\bigr\vert ^{2}\frac {1-|z_{n}|^{2}}{1-|\varphi_{1}(z_{n})|^{2}}>0, $$

because

$$\frac{1-|z_{n}|^{2}}{1-|\varphi_{1}(z_{n})|^{2}}\geq\frac{1-\varepsilon }{1+\varepsilon}\frac{1-|z_{n}|^{2}}{1-|\varphi_{2}(z_{n})|^{2}} $$

by $z_{n} \in H_{2}$ and (2.3). Thus we also have $\eta\in F_{u_{1}}(\varphi_{1})$. This leads to a contradiction to (3.8). So our claim holds. Thus by Lemma 2.1, we have

$$\sum_{i=1}^{i=2} \int_{ H_{2}}\vert u_{i}\vert ^{2}\bigl\vert f_{n}(\varphi _{2})\bigr\vert ^{2}\,d \lambda_{\alpha} =\sum_{i=1}^{i=2} \int_{ \mathbf{D} }\vert \chi _{H_{2}}u_{i}\vert ^{2}\bigl\vert f_{n}(\varphi_{2})\bigr\vert ^{2}\,d\lambda_{\alpha} \rightarrow0 $$

as $n\rightarrow\infty$. Therefore the proof is complete. □

The following, given in [27] and [1], respectively, are immediate consequences of Theorems 3.2 and 3.4.

### Corollary 3.5

*Let* $\varphi_{1}$, $\varphi_{2}$ *be holomorphic self*-*maps of* **D** *and* *a*, *b* *non*-*zero constants*. *If* $F(\varphi_{1})\neq\emptyset$ *and* $F(\varphi_{2})\neq\emptyset$, *then* $aC_{\varphi_{1}}+bC_{\varphi_{2}}$ *is compact on* $A^{2}_{\alpha}$ *if and only if* $a+b=0$ *and* $\lim_{|z|\rightarrow1}\rho(z) (\frac {1-|z|^{2}}{1-|\varphi_{1}(z)|^{2}}+\frac{1-| z|^{2}}{1-| \varphi _{2}(z)|^{2}} )=0$.

### Corollary 3.6

*Let* $\varphi_{1}$, $\varphi_{2}$ *be holomorphic self*-*maps of* **D**, *then* $C_{\varphi_{1}}-C_{\varphi_{2}}$ *is compact on* $A^{2}_{\alpha}$ *if and only if* $\lim_{| z|\rightarrow1}\rho(z) (\frac {1-|z|^{2}}{1-|\varphi_{1}(z)|^{2}}+\frac{1-| z|^{2}}{1-| \varphi _{2}(z)|^{2}} )=0$.

### Corollary 3.7

*Let* $u_{1}$, $u_{2}$ *be bounded holomorphic functions on* **D**, *then* $M_{u_{1}}-M_{u_{2}}$ *is compact on* $A^{2}_{\alpha}$ *if and only if* $u_{1}=u_{2}$.

## Compact perturbation

In the final section, we consider the compact perturbation of finite summations of a given weighted composition operator.

### Theorem 4.1

*For* $i=1,2,\ldots,N$, *let* $\varphi,\varphi_{i}\in\mathbf{S}(\mathbf {D})$ *and* *u*, $u_{i}$ *bounded holomorphic functions on* **D**. *Suppose that* $F_{u_{i}}(\varphi_{i}) \neq\emptyset$ *for each* *i*, $F_{u_{i}}(\varphi_{i})\cap F_{u_{j}}(\varphi_{j})=\emptyset$ ($i\neq j$), $F_{u}(\varphi)=\bigcup^{N}_{i=1}F_{u_{i}}(\varphi_{i})$. *Define* $\rho_{i}(z):=\vert \frac{\varphi(z)-\varphi_{i}(z)}{1-\overline {\varphi_{i}(z)}\varphi(z)}\vert $. *If*

1. $\lim_{z\rightarrow\xi}\rho_{i}(z) (|u(z)|^{2}\frac {1-|z|^{2}}{1-|\varphi(z)|^{2}}+|u_{i}(z)|^{2}\frac{1-| z|^{2}}{1-| \varphi_{i}(z)|^{2}} )=0$, *and*
2. $\lim_{z\rightarrow\xi}|u(z)-u_{i}(z)|=0 $ *for any* $\xi\in F_{u_{i}}(\varphi_{i})$

*for every* $i=1,2,\ldots, N$, *then* $uC_{\varphi}-\sum_{i=1}^{N}u_{i}C_{\varphi_{i}}$ *is compact on* $A_{\alpha}^{2}$.

### Proof

Define $\mathbf{D}_{i}:= \{z\in\mathbf{D}:|u_{i}(z)|^{2}\frac {1-|z|^{2}}{1-|\varphi_{i}(z)|^{2}}\geq|u_{j}(z)|^{2}\frac {1-|z|^{2}}{1-|\varphi_{j}(z)|^{2}}, \text{for all } j\neq i \}$ for each $i=1,2,\ldots,N$. Fix $\varepsilon>0$ and denote $E_{i}:=\{z\in \mathbf{D}_{i}, \rho_{i}(z)\leq\varepsilon\}$ and $E'_{i}:=\mathbf {D}_{i}\backslash E_{i}$. To end the proof, we assume that $\{f_{n}\}$ is any bounded sequence in $A^{2}_{\alpha}$ such that $f_{n}\rightarrow0$ ($n\rightarrow\infty$) uniformly on each compact subset of **D**. For $1\leq i\leq N$,

4.1 $$\begin{aligned}& \int_{\mathbf{D}_{i}}\Biggl\vert uf_{n}(\varphi)-\sum _{k=1}^{N}u_{k}f_{n}( \varphi_{k})\Biggr\vert ^{2}\, d\lambda_{\alpha} \\& \quad \lesssim \int_{\mathbf{D}_{i}}\bigl\vert uf_{n}(\varphi)-u_{i}f_{n}( \varphi _{i})\bigr\vert ^{2}\, d\lambda_{\alpha}+\sum _{k\neq i} \int_{\mathbf {D}_{i}}\bigl\vert u_{k}f_{n}( \varphi_{k})\bigr\vert ^{2}\, d\lambda_{\alpha} \\& \quad \lesssim \int_{E_{i}}\bigl\vert uf_{n}(\varphi)-u_{i}f_{n}( \varphi_{i})\bigr\vert ^{2}\, d\lambda _{\alpha}+ \int_{E'_{i}}\bigl\vert uf_{n}(\varphi)\bigr\vert ^{2}\, d\lambda_{\alpha} \\& \qquad {} + \int_{E'_{i}}\bigl\vert u_{i}f_{n}( \varphi_{i})\bigr\vert ^{2}\, d\lambda_{\alpha}+ \sum _{k\neq i} \int_{\mathbf{D}_{i}}\bigl\vert u_{k}f_{n}( \varphi_{k})\bigr\vert ^{2}\, d\lambda _{\alpha}. \end{aligned}$$

Let $\chi_{\mathbf{D}_{i}}$ and $\chi_{E'_{i}}$ be the characteristic functions of $\mathbf{D}_{i}$ and $E'_{i}$, respectively, then it is obvious from the assumption (1) that

4.2 $$ \lim_{|z|\rightarrow1}\chi_{E'_{i}} \biggl(\bigl\vert u_{i}(z)\bigr\vert ^{2}\frac {1-|z|^{2}}{1-|\varphi_{i}(z)|^{2}}+\bigl\vert u(z)\bigr\vert ^{2}\frac {1-|z|^{2}}{1-|\varphi(z)|^{2}} \biggr)=0. $$

Moreover,

4.3 $$ \lim_{|z|\rightarrow1}\chi_{\mathbf{D}_{i}}\bigl\vert u_{k}(z)\bigr\vert ^{2}\frac {1-|z|^{2}}{1-|\varphi_{k}(z)|^{2}}=0 $$

for fixed *i* and each $k\neq i$. Indeed, if (4.3) fails for some $k\neq i$, then there exist $\xi \in\mathbf{T}$ and $z_{n}\in\mathbf{D}_{i}$ satisfying $z_{n}\rightarrow\xi$ such that

$$\lim_{n\rightarrow\infty}\bigl\vert u_{k}(z_{n})\bigr\vert ^{2}\frac {1-|z_{n}|^{2}}{1-|\varphi_{k} (z_{n})|^{2}}>0. $$

Then $\xi\in F_{u_{k}}(\varphi_{k})$ and

$$\lim_{n\rightarrow\infty}\bigl\vert u_{i}(z_{n})\bigr\vert ^{2}\frac {1-|z_{n}|^{2}}{1-|\varphi_{i}(z_{n})|^{2} }>0 $$

by the definition of $\mathbf{D}_{i}$, which implies $\xi\in F_{u_{i}}(\varphi_{i})$. So $F_{u_{i}}(\varphi_{i})\cap F_{u_{k}}(\varphi_{k})\neq\emptyset$ when $i\neq k$, which contradicts our assumption. Then the last three terms of (4.1) tend to 0 as $n\rightarrow\infty $ by Lemma 2.1. In the following, we consider the first term of (4.1) by a similar argument to the proof of Theorem 3.4. For any $\xi\in F_{u_{i}}(\varphi_{i})$, there exists $\delta(\xi)>0 $ such that

$$\bigl\vert u(z)-u_{i}(z)\bigr\vert < \varepsilon, $$

whenever $|z-\xi|<\delta(\xi)$. We decompose $E_{i}$ into two parts as $E_{i}=H_{i1}+H_{i2}$, where

$$H_{i1}:=E_{i}\cap \biggl(\bigcup _{\xi\in F_{u}(\varphi)}\bigl\{ z\in{\mathbf {D}}:|z-\xi|< \delta(\xi)\bigr\} \biggr) $$

and $H_{i2}:=E_{i}\backslash H_{i1}$. Note that

4.4 $$\begin{aligned}& \int_{E_{i}}\bigl\vert uf_{n}(\varphi)-u_{i}f_{n}( \varphi_{i})\bigr\vert ^{2}\, d\lambda _{\alpha} \\& \quad \lesssim \int_{E_{i}}\bigl\vert f_{n}(\varphi)-f_{n}( \varphi_{i})\bigr\vert ^{2}\, d\lambda_{\alpha }+ \int_{E_{i}}\vert u-u_{i}\vert ^{2}\bigl\vert f_{n}(\varphi_{i})\bigr\vert ^{2}\, d \lambda_{\alpha} \\& \quad \lesssim \int_{E_{i}}\bigl\vert f_{n}(\varphi)-f_{n}( \varphi_{i})\bigr\vert ^{2}\, d\lambda_{\alpha }+\sum ^{j=2}_{j=1} \int_{H_{ij}}\vert u-u_{i}\vert ^{2}\bigl\vert f_{n}(\varphi _{i})\bigr\vert ^{2}\, d \lambda_{\alpha} \\& \quad \lesssim \int_{E_{i}}\bigl\vert f_{n}(\varphi)-f_{n}( \varphi_{i})\bigr\vert ^{2}\, d\lambda_{\alpha }+ \int_{H_{i1}}\vert u-u_{i}\vert ^{2}\bigl\vert f_{n}(\varphi_{i})\bigr\vert ^{2}\, d \lambda_{\alpha} \\& \qquad {}+ \int_{H_{i2}}\vert u\vert ^{2}\bigl\vert f_{n}(\varphi_{i})\bigr\vert ^{2}\, d \lambda_{\alpha}+ \int _{H_{i2}}\vert u_{i}\vert ^{2}\bigl\vert f_{n}(\varphi_{i})\bigr\vert ^{2}\, d \lambda_{\alpha}. \end{aligned}$$

Clearly, by (3.5)

$$\int_{E_{i}}\bigl\vert f_{n}(\varphi)-f_{n}( \varphi_{i})\bigr\vert ^{2}\, d\lambda_{\alpha}\leq \int_{\{z\in\mathbf{D}: \rho_{i}(z)\leq\varepsilon\}}\bigl\vert f_{n}(\varphi )-f_{n}(\varphi_{i})\bigr\vert ^{2}\, d \lambda_{\alpha}\lesssim\varepsilon^{2} $$

for all *n*. Also, by the definition of $H_{i1}$, we can easily get

$$\int_{ H_{i1}}\vert u-u_{i}\vert ^{2}\bigl\vert f_{n}(\varphi_{i})\bigr\vert ^{2}\, d \lambda_{\alpha }\leq\varepsilon^{2}\Vert C_{\varphi_{i}}f_{n} \Vert ^{2}_{A^{2}_{\alpha }}\lesssim\varepsilon^{2} $$

for all *n*. Moreover,

4.5 $$ \lim_{|z|\rightarrow1}\chi_{H_{i2}}\bigl\vert u(z) \bigr\vert ^{2}\frac{1-|z|^{2}}{1-|\varphi (z)|^{2}}=0. $$

Indeed, if (4.5) fails, there exist some $\zeta\in\mathbf{T}$ and a sequence $\{z_{n}\}\subseteq H_{i2}$ satisfying $z_{n}\rightarrow\zeta$ such that

$$ \lim_{|z|\rightarrow1}\bigl\vert u(z)\bigr\vert ^{2} \frac{1-|z|^{2}}{1-|\varphi(z)|^{2}}>0, $$

then $\zeta\in F_{u}(\varphi)=\bigcup^{N}_{k=1}F_{u_{k}}(\varphi_{k})$. Since this $\zeta\notin F_{u_{k}}(\varphi_{k})$ when $k\neq i$ by (4.3), then $\zeta\in F_{u_{i}}(\varphi_{i})$, which contradicts the definition of $H_{i2}$. So (4.5) holds. We now claim that

4.6 $$ \lim_{|z|\rightarrow1}\chi_{H_{i2}}\bigl\vert u_{i}(z)\bigr\vert ^{2}\frac {1-|z|^{2}}{1-|\varphi_{i}(z)|^{2}}=0 $$

and

4.7 $$ \lim_{|z|\rightarrow1}\chi_{H_{i2}}\bigl\vert u(z) \bigr\vert ^{2}\frac{1-|z|^{2}}{1-|\varphi _{i}(z)|^{2}}=0. $$

Indeed, the argument for (4.6) is similar to (3.9) and we omit it. To prove that (4.7) holds, we assume that there exist some $\eta\in\mathbf{T}$ and a sequence $\{z_{n}\}\subseteq H_{i2}$ such that $z_{n}\rightarrow\eta$ and

$$\lim_{n\rightarrow\infty}\bigl\vert u(z_{n})\bigr\vert ^{2}\frac{1-|z_{n}|^{2}}{1-|\varphi _{i}(z_{n})|^{2}}>0. $$

Note that

$$\frac{1-|z_{n}|^{2}}{1-|\varphi(z_{n})|^{2}}\geq\frac{1-\varepsilon }{1+\varepsilon}\frac{1-|z_{n}|^{2}}{1-|\varphi_{i}(z_{n})|^{2}}, $$

because of $\{ z_{n} \}\subseteq H_{i2}$ and (2.3). Then $\eta\in F_{u}(\varphi)$, which contradicts (4.5). Thus again by Lemma 2.1, we have

$$\int_{H_{i2}}\vert u_{i}\vert ^{2}\bigl\vert f_{n}(\varphi_{i})\bigr\vert ^{2}\, d \lambda_{\alpha}+ \int _{H_{i2}}\vert u\vert ^{2}\bigl\vert f_{n}(\varphi_{i})\bigr\vert ^{2}\, d \lambda_{\alpha}\rightarrow0 $$

as $n\rightarrow\infty$. So

$$\int_{\mathbf{D}_{i}}\Biggl\vert uf_{n}(\varphi)-\sum _{k=1}^{N}u_{k}f_{n}( \varphi_{k})\Biggr\vert ^{2}\, d\lambda_{\alpha} \rightarrow0, $$

and then

$$\Biggl\Vert \Biggl(uC_{\varphi}-\sum_{i=1}^{N}u_{i}C_{\varphi_{i}} \Biggr)f_{n}\Biggr\Vert ^{2}_{A^{2}_{\alpha}}\lesssim\sum _{i=1}^{N} \int_{\mathbf {D}_{i}}\Biggl\vert uf_{n}(\varphi)-\sum _{k=1}^{N}u_{k}f_{n}( \varphi_{k}) \Biggr\vert ^{2}\, d\lambda_{\alpha} \rightarrow0 $$

as $n\rightarrow\infty$, which completes the proof. □
